# The extent of western lowland gorilla social relationships within and between groups

**DOI:** 10.1371/journal.pone.0316598

**Published:** 2025-01-24

**Authors:** Kristena Cooksey, Jake Funkhouser, Crickette Sanz, Jean Marie Massamba, Thierry Fabrice Ebombi, Prospère Teberd, Gaston Abea, Gaeton Mbebouti, Kathryn Judson, Sean Brogan, Colleen Stephens, David Morgan

**Affiliations:** 1 Department of Anthropology, Washington University in Saint Louis, Saint Louis, Missouri, United States of America; 2 Department of Language & Cultural Studies, Anthropology and Sociology, Eastern Kentucky University, Richmond, Kentucky, United States of America; 3 Department of Evolutionary Anthropology, University of Zurich, Zurich, Switzerland; 4 Wildlife Conservation Society, Congo Program, Brazzaville, Republic of Congo; 5 Fisher Center for the Study and Conservation of Apes, Lincoln Park Zoo, Chicago, Illinois, United States of America; Universidad de Guadalajara, MEXICO

## Abstract

The nature of western lowland gorilla social relationships within and between groups is largely understudied, partly due to the challenges of monitoring associations between individuals who live in neighboring groups. In this study, we examined the social relationships of four western lowland gorilla groups in the Ndoki landscape of northern Republic of Congo. To do so, we compiled all-occurrence social interaction and silverback nearest neighbor social networks from data collected during daily group follows conducted over several years. We observed a total of 5,923 dyadic all-occurrence social interactions (1,350 ± 489 per group, 138 intergroup interactions) and 54,989 dyadic silverback nearest neighbor associations (13,747 ± 3,963 observations per group, 105 nearest neighbor observations of intergroup partners during group scans). For all groups, we found that males were more social than females, younger individuals were more social than older gorillas, and slightly greater rates of social behaviors were observed during periods of higher fruit availability. While there was a considerable amount of interindividual variation in social behavior, the network of social interactions demonstrated a large extent of social relationships within and between groups. Additionally, we performed simulated network removals to assess the impact on social dynamics. Across all groups and the total population, the removal of blackback and immature individuals markedly decreased the number of intra- and intergroup relationships (>60% decrease). The documented extent of western lowland gorilla social relationships has direct implications for the conservation of species with multi-level social dynamics. Gaining clarity on the ways in which western lowland gorilla groups naturally occur in the wild, not only provides a greater understanding for their conservation, but also offers insights for managing their social dynamics within captive environments.

## Introduction

Longitudinal studies of gorilla behavior and ecology have revealed unexpected variability across almost all dimensions of their socioecology [1]. While little to no competition for abundant herbaceous vegetation exists within or between overlapping multimale-multifemale mountain gorilla groups (*Gorilla beringei beringei*, [2]), the same is not predicted for western lowland gorillas (*Gorilla gorilla gorilla*, [3]). Western lowland gorillas traverse diverse and heterogeneous habitats in the pursuit of ripe fruits and preferred nutrient-rich aquatic plants [3–5]. The breeding groups of this species are smaller than their mountain counterparts and historically have been described to be predominantly composed of a single silverback male, multiple adult females, and their dependent offspring [6–8]. Even though longitudinal investigations of western lowland gorilla social behavior are absent in the literature, there is evidence suggesting that the distribution and availability of fruits influences some aspects of their ranging, activity patterns, and transmission of information and pathogens [9–14]. While there is much to be learned about the potential factors that drive variation in social behavior (e.g., resource availability, risk of disease) of western lowland gorillas, the overall patterning and extent of their social relationships within and across groups still remains to be examined.

### Intergroup social behavior in western lowland gorillas

It has been predicted that frugivorous western lowland gorillas aggressively interact with neighboring groups in competition for preferred fruit items [15, 16]. However, both direct observations and genetic evidence of western lowland gorilla intergroup encounters describe a surprising paucity of agonistic opposition [17–20]. Rather than being characterized as entirely aggressive, western lowland gorilla intergroup encounters are often tolerant or avoided. For example, when groups visit fruit-bearing trees in the periphery of their home ranges they avoid opportunities to co-feed, rather than compete for access to fruit [21]. Initial genetic surveys of unhabituated western lowland gorilla groups revealed that silverbacks were usually related to one or more nearby, neighboring males. Therefore, patrilocal dispersal patterns have been hypothesized to contribute to an extended network of familiar individuals [17]. Subsequent remote methods have noted less relatedness across unhabituated western lowland gorilla groups but revealed a surprising degree of fluidity in group structure, suggesting the acceptance of non-kin, young adult males to social units and play between intergroup immatures [19]. However, the possibility of durable, social relationships between members of neighboring groups, specifically silverbacks, have yet to be examined longitudinally via direct observations.

In the Nouabalé-Ndoki National Park (Republic of Congo), direct observational methods of monitoring neighboring habituated groups have deepened our understanding of several aspects of the socioecology of western lowland gorillas, including, but not limited to, the nature of intergroup encounters [18] and flexibility of home range overlap [22]. In this region, the home ranges of neighboring western lowland gorilla groups overlap by more than 50% on average [22]. Further, members of these groups are often tolerant of familiar males and engage in affiliative interactions with individuals from other groups on occasion. These groups even interacted (sometimes peacefully) with solitary males who had previously dispersed from their groups [18]. However, it is unclear if these types of tolerant and affiliative western lowland gorilla intergroup encounters could potentially scaffold into the repeated associations and interactions necessary to construct individually recognizable, and familiar social relationships [23–25].

Reports of tolerant western lowland gorilla intergroup encounters further promote the possibility of dispersed communities where individuals are familiar with or related to individuals in other groups [17, 19, 26, 27]. Further, it has been suggested that this pattern of social structure may represent multi-level societies with groups nested within communities ([20], also see [28, 29]). Additionally, the average spatial spread of individuals in western lowland gorilla groups is much higher than what is typical of mountain gorillas [4, 9, 30], and thus western lowland individuals are likely able to make much more salient choices about with whom to spend their time and socially interact. Such flexibility in group spread even allows individual gorillas to form unique patterns of social relationships with sympatric chimpanzees, as reported from the Nouabalé-Ndoki National Park [31]. Therefore, it is possible that western lowland gorilla social relationships may also permeate across neighboring gorilla groups.

### Intragroup social behavior in western lowland gorillas

Daily follows of the full spectrum of gorilla social behavior that can be observed as they traverse different forest habitats (including visits to clearings, referred to as “bais”) and encounter other groups provide the opportunity to examine patterns of social relationships within and between groups. Gorilla intragroup social behavior has largely been described as centering around the silverback male who fosters the greatest protection against events of possible predation and infanticide [6, 32]. Tolerant relationships typically exist among silverbacks, established adult females, and immatures in the group [33–35]. Immature individuals of both sexes frequently play with their peers and may also include young adult males and the silverbacks in these interactions [9, 19]. Although gorillas display overall low rates of overt affiliative interactions, the strongest relationships (aside from those involving immature individuals) occur between adult males and new immigrant females [36]. However, aggressive interactions also regularly occur in this social context [33]. Gaining a better understanding of the different types of relationships between adult western lowland gorillas is especially critical given the frequent contrasts made to their mountain gorilla counterparts. To provide another example, there are mixed reports of multimale groups in western lowland gorilla society [8, 21, 37–40]. It has been suggested that western lowland groups contain multiple silverback males for less than two years [8], with any insights into the relationships within these social units still forthcoming. A summary of previous investigations related to western lowland gorilla social behavior is provided in *Table 1*. While western lowland gorilla social organization has been described from bai observations and with genetic assessments, our understanding of the social relationships in western lowland gorilla groups is limited, especially with regards to social interactions between members of different age-sex classes.

**Table 1:** Overview of western lowland gorilla social behavior.

*Study Topic*: Study Site	Methods					Summary of relevant results
***Social Structure***:						
Mondika [17]	G					Familiar adult male kin frequently ranged in proximity to one another to form extended male networks
Mondika [27], Loango [26]	G					Adult female kin frequently shared group membership
Mbeli Bai [20]		B				Patterns of tolerant and avoidant intergroup encounters indicated nested structure of one-male groups within a community of neighboring groups
Mbeli Bai [6, 38], Maya Bai [37]		B				Most groups were composed of one silverback male, no evidence of groups with more than one silverback male and no evidence of subgrouping
Bai Hokou, Lossi, Maya Bai, Mbeli Bai [8]		B		F	N	Groups typically composed of one silverback male, brief durations of multimale groups observed prior to the dispersal of young silverbacks
Odzala-Kokoua [19]			C	F	N	Groups interacted frequently and non-aggressively to form communities
***Intergroup Encounters***:						
Bai Hokou, Mongambe [41]			W			Lethal intraspecies encounters may occur, albeit rarely
Lossi [21]				F		Intergroup encounters were tolerant or avoided, sometimes forming overnight nesting supergroups
Mondika, Goualougo [18]				F	N	Most intergroup encounters were tolerant or avoided
***Ranging***:						
Mondika [30]				F		Ranging behavior was not affected by encounters with neighboring groups
Mondika, Goualougo [22]				F	N	Intergroup overlap in ranging patterns was variable, but overlap was consistent
***Social Relationships***:						
Mbeli Bai [32, 42]		B				Male and females shared strong social relationships
Mbeli Bai [33]		B				Affiliation was rare overall, male-female interactions could be agonistic
Bai Hokou [9]				F		Availability of fruit impacted proportion of social behavior in activity patterns, play involving immature individuals was common
Mondika, Goualougo [*This study*]				F	N	Considerable extent of tolerant, affinitive social relationships within and between groups

*Methods*: [G] Genetic surveys; [B] Observations in bais (i.e., forest clearings); [W] Wounding patterns observed on deceased individuals; [C] Camera trap observations; [F] Daily follows of focal groups; [N] Daily follows of neighboring groups.

We aimed to investigate patterns of social behavior of four western lowland gorilla groups in northern Republic of Congo across eight years of direct observations. Specifically, we examined the potential factors that might influence patterns of western lowland gorilla social behavior (e.g., the availability of food resources) and assessed the extent of social relationships within and between groups. Following previous lines of inquiry, we specifically predicted (*a*) rates of social behavior to increase with fruit availability [11]; (*b*) social relationships to extend across neighboring groups, including between neighboring silverback males [17, 19, 20, 27]; and (*c*) intragroup social relationships to be centered around the silverback males [9, 17, 36]. Another important aim of this research is to relate our findings to the conservation of these endangered apes.

## Methods

### Study sites and subjects

**Fig 1:**
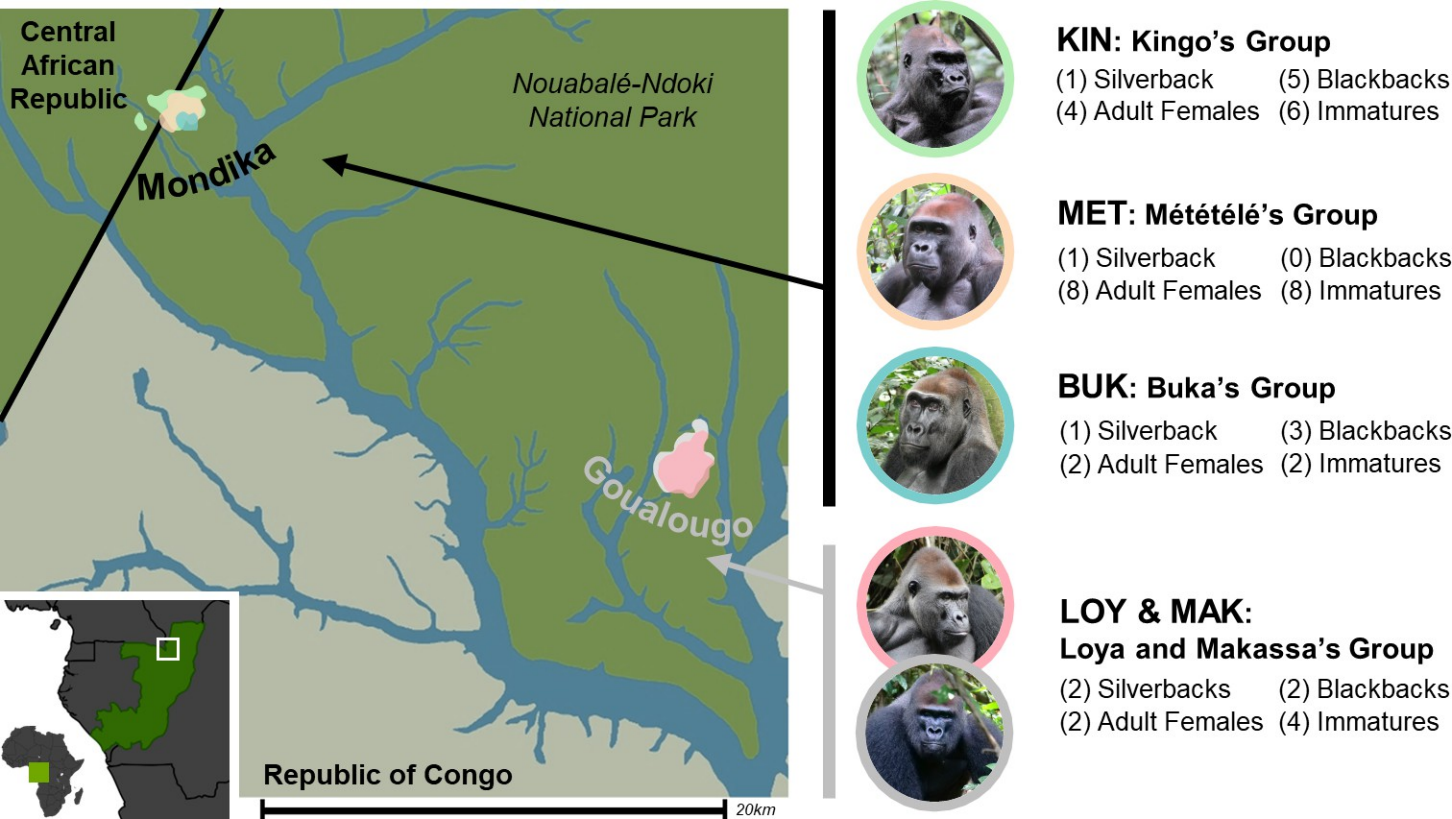
Demographics and home ranges of the study groups within and along the periphery of the Nouabalé-Ndoki National Park (NNNP), Republic of Congo. Approximations of each group’s home ranges have been drawn from [22]. Group composition values represent the total number of unique individuals for each age-sex class in each group across all eight years of data collection. Immatures include infant and juvenile individuals. Only one female transferred between study groups: Kenga is counted as an immature in her natal group (Kingo group) and an adult female in Mététélé’s group. Six males were observed across their immature and blackback ages, and all six are counted as blackback males given their average age during the study period. The components of this figure are either property of the authors, have been drawn from materials in the public domain [44, 45], or were created with BioRender.com (reprinted under a CC BY license, original copyright (2024)).

This study focused on four habituated groups of western lowland gorillas (*Gorilla gorilla gorilla*) in the Nouabalé-Ndoki National Park, Republic of Congo. We studied the neighboring Kingo, Buka, and Mététélé groups at the Mondika research station and the Loya-Makassa group, located in the Goualougo Triangle (see *Fig 1*). Kingo, Buka, and Loya-Makassa groups were habituated prior to the study period of this investigation, Mététélé’s group was habituated in 2018 and data collection initiated thereafter [43]. Mondika is located in the Djéké Triangle, which was incorporated into the Nouabalé-Ndoki National Park in 2023, and is bordered to the east by Dzanga–Ndoki National Park in the Central African Republic (mean annual rainfall = 1915.3 mm, SD = 107.5; mean annual temperatures = 21.6°C, SD = 0.3°C to mean = 27.3°C, SD = 0.9°C). The Goualougo Triangle is located in the southernmost portion of the Nouabalé-Ndoki National Park (mean annual rainfall = 1714.3 mm, SD = 177.1; mean annual temperatures = 21.4°C, SD = 0.2°C to mean = 24.8°C, SD = 1.6°C). This study includes 8 years of data between 2014–2021.

The Kingo, Buka, Mététélé, and Loya-Makassa groups ranged in size from 5 to 17 gorillas (mean = 11.4 individuals, SD = 3.9) with 1–2 silverbacks, 2–5 blackbacks (mean = 3.0, SD = 1.2), 1–8 adult females (mean = 3.6, SD = 2.6), and 1–3 immature individuals (i.e., infants and juveniles: mean = 1.8, SD = 0.8) (see *Fig 1*). During August 2020 in Goualougo, Loya group was joined by extra-group silverback Makassa. They remained together and continued to range as a multi-male group. Additionally, in January 2019 at Mondika, one adult female gorilla (Kenga) transferred from her natal group (Kingo) to neighboring Mététélé’s group. In total, N = 51 individuals were included in data collection during this study period.

### Behavioral data collection

Research assistants for the Goualougo Triangle Ape Project and Mondika Gorilla Project conducted daily group follows for each study group. All occurrences of social interactions between gorillas (i.e., play, groom, aggression, submission, reproductive, food share, beg, nurse, approach, follow) were recorded at each observation and instantaneous 20-minute group scan observations were conducted throughout each day to record the behavior of all individuals and the nearest neighbor to the silverback. All observations were recorded using a custom configuration of the Animal Observer App (Version 1.4). All daily group follows and behavioral data collection were conducted using current best practices for minimizing any possible spread of anthropozoonotic pathogens to wild great ape populations [46].

### Generalized linear mixed model

To test for the influence of fruit availability on patterns of social behavior, fruit availability was calculated from the proportion of monitored trees fruiting in the Goualougo Triangle [18]. We examined variation in the proportion of instantaneous 20-minute group scan records with observed social behavior using a generalized linear mixed model with negative binomial error structure and log link function. The model was run in R (version 4.0.3) using glmmTMB [47]. All four of the groups were considered for the model (Buka, Kingo, Loya-Makassa, and Mététélé). As the response variable, we used the number of group scan observations where a given individual was observed to be engaging in social behavior for each month through the study period (mean = 7.88, SD = 12.80). In this model, we only included data for individuals for which there were at least five months of data, with each month having at least 20 total scans. A total of 1,419 data points were included in this model. Our test predictors (i.e., the predictors of interest) were sex (females = 463, males = 956), age (mean = 17.60 years, SD = 11.97), group size (mean = 7.84 individuals, SD = 3.11), proportion of immatures (mean = 0.28 individuals, SD = 0.11), fruit availability (mean = 0.36 fruiting trees, SD = 0.11), and rainfall (mean = 145.44 cm, SD = 99.96). We also included group identity as a control predictor (Buka N = 359, Kingo N = 565, Loya-Makassa N = 384, Mététélé N = 111), as well as the log-transformed number of total monthly scans as an offset term. The ID of the individual (N = 38) was included as a random intercept [48] as were random slopes of age, group size, proportion of immatures, fruit availability, and rainfall [49, 50]. We z-transformed all continuous predictors to a mean of 0 and a standard deviation of 1. We determined there was no concern regarding multicollinearity by calculating variance inflation factors for the fixed effects; maximum VIF = 2.89 [51]. We also reiteratively analyzed the model removing data points from each individual sequentially to assess the stability of the model. To establish the significance of the test predictors collectively, we first compared the full model, containing all fixed and random effects, to a reduced model, omitting the test predictors, using a likelihood ratio test [52]. Further tests of individual predictors were also performed using likelihood ratio tests.

### Social network analysis

We used social network analysis to examine the extent and connectivity of inter- and intragroup social relationships. Social network analysis provides quantitative measures that can be standardized for comparing social connectivity at the individual and group level across groups [53]. First, we constructed a network of social interactions within and between groups by compiling all observed social interactions for all groups across the entire study period. The social interaction network was calculated using a matrix of bi-directional total counts of social interactions for each gorilla dyad. Second, we constructed a network of silverback nearest neighbor associations by compiling observations of the nearest neighbor to each group’s silverback during instantaneous 20-minute group scans. The silverback nearest neighbor network was calculated using a matrix of simple ratio association indexes (i.e., the proportion of observations where X and Y were nearest neighbors relative to the number of observations of X and Y independently [54]). Any association indexes ≤0.0009 for any between-group dyads were rounded to 0.001 in the silverback nearest neighbor network. All individuals who resided in any of the study groups at the beginning of data collection (2014, N = 35), those who were born in any of the study groups during the study period (2014–2021, N = 12), and individuals who immigrated into any of the study groups during the study period (2014–2021, N = 5) were included in the social network analyses [19]. All individuals resided in one of the study groups for at least one year of data collection and were observed to be the silverback’s nearest neighbor or engage in a social interaction at least once during the study period. Given the longitudinal nature of our dataset, we assigned individuals to age classes for the social network analyses based on their average age during our study period [55]. All infant and juvenile individuals were categorized as immature. During this time, six male gorillas were observed across immature and blackback ages (Buka group: FNN and PAK; Kingo group: EKE, ITE, and KUS; Loya group: MOD). All six males were categorized as blackback males for social network visualizations and the simulated removal analyses based on their average ages during the study period. Also, during the study period, one female gorilla (Kenga) transferred from her natal group (Kingo) when she reached adulthood to neighboring Mététélé’s group. For this reason, Kenga (KEN) is represented as an immature individual in her natal group and an immigrant adult female in the group to which she dispersed (KEN2: Mététélé). We constructed the visual rendering for both networks using the graphopt layout algorithm in iGraph 1.2.4.1 in R, where individuals (nodes) with the strongest relationships (edges) are plotted near one another, and individuals with the highest network centrality are plotted in the group’s center. We manually adjusted node placements to minimize node overlap and intersecting edges [56].

To test the extent of social relationships across neighboring groups, we constructed an expected social interaction network for null hypothesis significance testing. Following previously published records of wild western lowland gorilla intergroup social behavior (e.g., [17, 19, 20, 27]), the expected social interaction network consisted of binary edges (i.e., 1 = the presence a of social relationships, 0 = the absence of a social relationship) between all within-group individuals and the group’s silverback, between all adult females and her offspring, as well as between maternal siblings. To perform this comparison, we then transformed the observed social interaction network to a binary network where all dyads who were observed to interact more than once during the study period were assigned a weighted binary edge (1, the presence of a social relationship) and all other dyads were assigned 0 (the absence of a social relationship). We then tested for a difference in social interaction network density between the observed and expected presence of social relationships using matrix-based bootstrapped t-tests. While limited in its ability to account for the relative probability of any dyad to interact over an eight-year period, binary network comparisons are powerful in representing the baseline differences between the expected networks and null models of interactions that occur at random. Results that indicate non-random patterns of social relationships can then be further evaluated in subsequent, detailed analyses of all-occurrence social interactions [54].

To further evaluate the influence of silverbacks and individuals in other age-sex classes on the observed patterns of social relationships, we conducted simulated removals for all silverbacks, blackbacks, adult females, and immature individuals for comparison of connectedness across observed and expected networks [55]. Simulated removals are commonly performed in the study of animal social networks as they are powerful in determining the relative importance of sets of individuals in maintaining the community’s social structure (i.e., network connectivity) while making no assumptions about the distribution of data nor the mode of data collection [57]. To perform these tests, we constructed simulated removal matrixes where the observed social interactions and nearest neighbor association index values (i.e., edges) were replaced with zeros for all individuals of a given age-sex class in each respective simulated removal matrix. For simulated removal analyses, we included KEN as an immature individual, KEN2 as an adult female, as well as FNN, PAK, EKE, ITE, KUS, and MOD as blackback males based on their average ages during the study period [55]. We then tested for differences between the simulated removal matrix for each age-sex class against the observed network of social interactions using matrix-based bootstrapped t-tests.

#### Matrix-based bootstrapped t-tests

We used matrix-based bootstrapped t-test analyses to compare the network density of (1) the observed binary social interaction networks with expected binary social interaction networks constructed from previously published records of wild western lowland gorilla intergroup social behavior (e.g., [17, 19, 20, 27]) and (2) the observed all-occurrence social interaction networks with simulated removal matrixes for all individuals in silverbacks, blackbacks, adult females, and immature (i.e., infants and juveniles) age-classes following standard procedures for these types of analyses [54, 58, 59]. Network density is an ideal metric for the comparison between observed and expected or simulated removal networks as it is an index of overall network connectedness (the proportion of observed to the possible total number of relationships between individuals) and can be calculated without bias for all-occurrence social interaction networks [25, 53, 60]. Importantly, these procedures make no assumptions about the distribution of the data nor the mode of data collection as it uses a bootstrapping procedure to permute the observed data to construct the t-test statistic [23, 57, 61, 62]. This test uses a bootstrapped t-test technique to compare the network connectivity across observed and expected or simulated removal networks against 5,000 randomly permuted networks that are constructed by randomly re-arranging, swapping, and shuffling the edges from the input matrixes to create a test statistic distribution for null significance hypothesis testing [63]. Our procedure mirrors the standard social network analyses of other authors who have been interested in similar questions (see [23, 54, 57–59, 64–67]). We conducted these tests in UCINET version 6.696 [59].

### Research ethics

This research was endorsed by the Institut National de Recherche Forestière which is part of the Ministère de l’Enseignement Supérieur, La Recherche Scientifique et de l’Innovation Technologique of the Republic of Congo. It was also approved by Institutional Animal Care and Use Committee of Washington University in St. Louis. We also confirm that all researchers associated with this study adhered to the Ethical Treatment of Nonhuman Primates outlined by the American Society of Primatologists.

## Results

From 2014–2021, we recorded a total of 5,923 social interactions (mean = 1,350, SD = 489 per group, 138 intergroup social interactions) and 54,989 dyadic silverback nearest neighbor associations (mean = 13,748, SD = 3,963 observations per group, 105 nearest neighbor observations of intergroup partners across a total of 3,673 group scans). Of all social interactions, 95% were observations of social play involving one or more immature individuals, 4% were other affiliative interactions, and less than 1% were aggressive interactions. Across all observations, only 8 aggressive intergroup social interactions were observed. *Table 2* provides a summary of observed social interactions and *Table 3* provides a summary of observed silverback nearest neighbor associations between gorillas.

**Table 2:** Observed frequencies of social interactions observed per dyads across age and sex classes.

	*N*	Play	Other Affiliation	Aggression
**Intragroup Partners**	***5*,*785***	***95%***	***4%***	***1%***
*Immature dyads*	3,249	99%	1%	0%
*Immature-Adult Female*	218	64%	34%	2%
*Immature-Blackback*	1,722	99%	1%	0%
*Immature-Silverback*	49	51%	39%	10%
*Adult Female dyads*	14	36%	29%	36%
*Adult Female-Blackback*	56	70%	21%	9%
*Adult Female-Silverback*	69	0%	90%	10%
*Blackback dyads*	395	97%	2%	1%
*Blackback-Silverback*	8	25%	38%	38%
*Silverback dyads*	5	20%	20%	60%
**Intergroup Partners**	***138***	***89%***	***5%***	***6%***
*Immature dyads*	25	100%	0%	0%
*Immature-Adult Female*	10	50%	40%	10%
*Immature-Blackback*	61	97%	2%	2%
*Immature-Silverback*	0	0%	0%	0%
*Adult Female-Blackback*	10	60%	20%	20%
*Adult Female-Silverback*	0	0%	0%	0%
*Blackback-Blackback*	29	97%	0%	3%
*Blackback-Silverback*	1	0%	0%	100%
*Silverback dyads*	2	0%	0%	100%

Play, other affiliation, and aggression social interaction observations were collected at every observation during daily group follows. (*N*) indicates total number of observed bidirectional dyadic social interactions, all other values indicate percent of total observed social interactions per dyad type.

**Table 3:** Observed frequency of silverback nearest neighbor associations observed per dyads across age and sex classes.

	*N*	Nearest Neighbor Associations
**Intragroup Partners**	**54,884**	**99%**
*Immature-Silverback*	*26*,*423*	48.05%
*Adult Female-Silverback*	*17*,*223*	31.32%
*Blackback-Silverback*	*10*,*865*	19.76%
*Silverback dyads*	*373*	0.68%
**Intergroup Partners**	**105**	**0.19%**
*Immature-Silverback*	3	0.01%
*Adult Female-Silverback*	95	0.18%
*Blackback-Silverback*	1	0.01%
*Silverback dyads*	6	0.01%

Silverback nearest neighbor associations were collected during instantaneous 20-minute group scans. (*N*) indicates total number of observed nearest neighbor associations, all other values indicate percent of observed associations per dyad type.

### Fruit availability and social behavior

**Fig 2:**
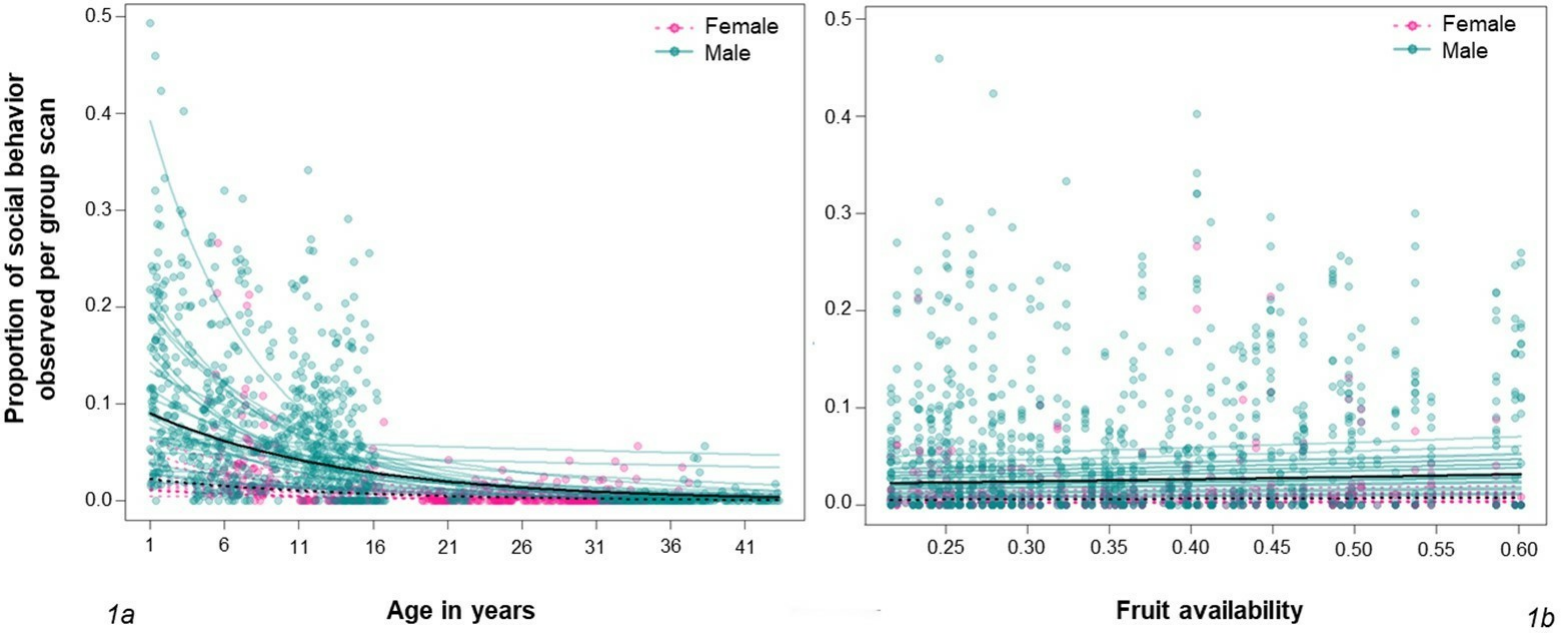
Effect of (a) Age and (b) Fruit Availability on Observed Gorilla Social Behavior. Each datapoint represents one month of data for a single individual and lines indicate the predicted proportion of social behavior at a given age for all males (solid black lines), all females (dotted black lines), and each individual (solid, semi-transparent lines).

The results of our GLMM analyses indicate that the prevalence of social behavior decreases with age, increases with fruit availability, and is more common in males (collective significance: *X*^*2*^ = 35.08, DF = 6, P < 0.01: *Table 4* and *Fig 2*). However, there was a considerable amount of interindividual variation in the observed frequency of social behavior, even among individuals of the same age-sex class. These results suggest that fruit availability has a relatively minor influence on western lowland gorilla social behavior and the influence of interindividual variation cannot be understated.

**Table 4:** Predictive factors of social behavior.

Factor	Estimate	SE	*X* ^*2*^	DF	P
Sex (male)	1.39	0.47	11.77	1	<0.01
Age	-0.90	0.25	6.43	1	0.01
Group Size	-0.16	0.16	1.03	1	0.31
Proportion of Immatures	0.14	0.08	2.88	1	0.09
Fruit Availability	0.09	0.03	7.97	1	<0.01
Rainfall	0.04	0.03	2.31	1	0.13
Group (Buka vs. Kingo)	0.51	0.45	*control*		
Group (Buka vs. Loya)	0.02	0.52	*control*		
Group (Buka vs. Mététélé)	-0.60	0.55	*control*		

### Intra- and intergroup social connectivity

**Fig 3:**
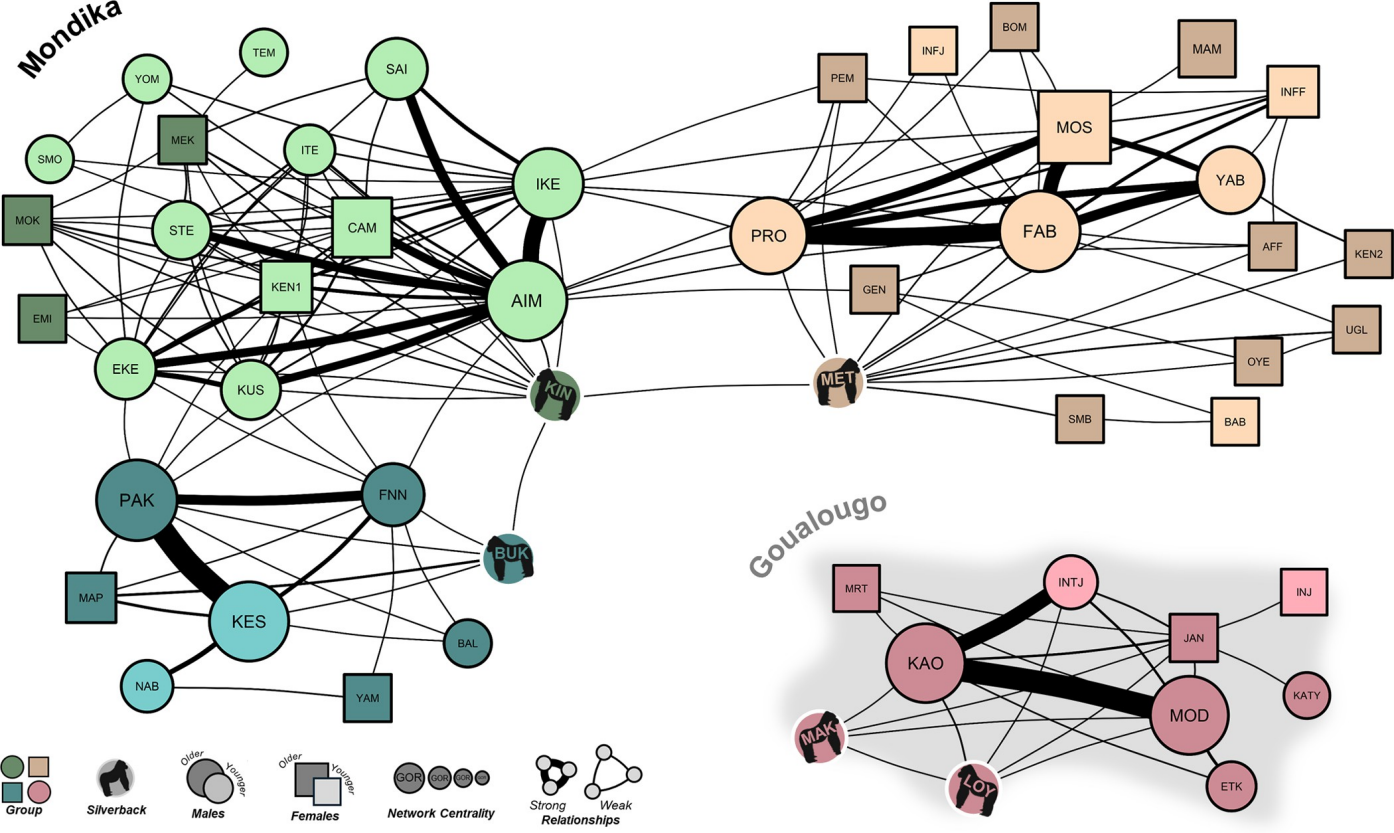
Social interaction networks for all study groups. Measured as the total counts of interactions between dyads. All shapes (nodes) represent individual gorillas and the lines connecting them represent the relationships that they share. The width of the lines is scaled to relationship strength (total number of interactions). The size of nodes and node labels (i.e., gorilla name) are scaled to within-group network centrality (an individual-level metric of network importance and influence: [60]), and the color of the node indicates group membership. The shape of the node indicates sex, where females and immature individuals of unknown sex are squares and males are circles. Nodes are shaded to indicate average age during the study period, where the darkest are adults (males >11 years, females >10 years) and the lightest nodes are immatures (i.e., infants and immatures). The silverback individuals are highlighted with gorilla silhouettes. The Loya-Makassa group resides in Goualougo (background shaded grey), whereas the neighboring Buka, Kingo, and Mététélé groups reside in Mondika. Silverback gorilla icons were created with BioRender.com and are reprinted under a CC BY license, with permission from BioRender, original copyright (2024), and used only for illustrative purposes.

**Fig 4:**
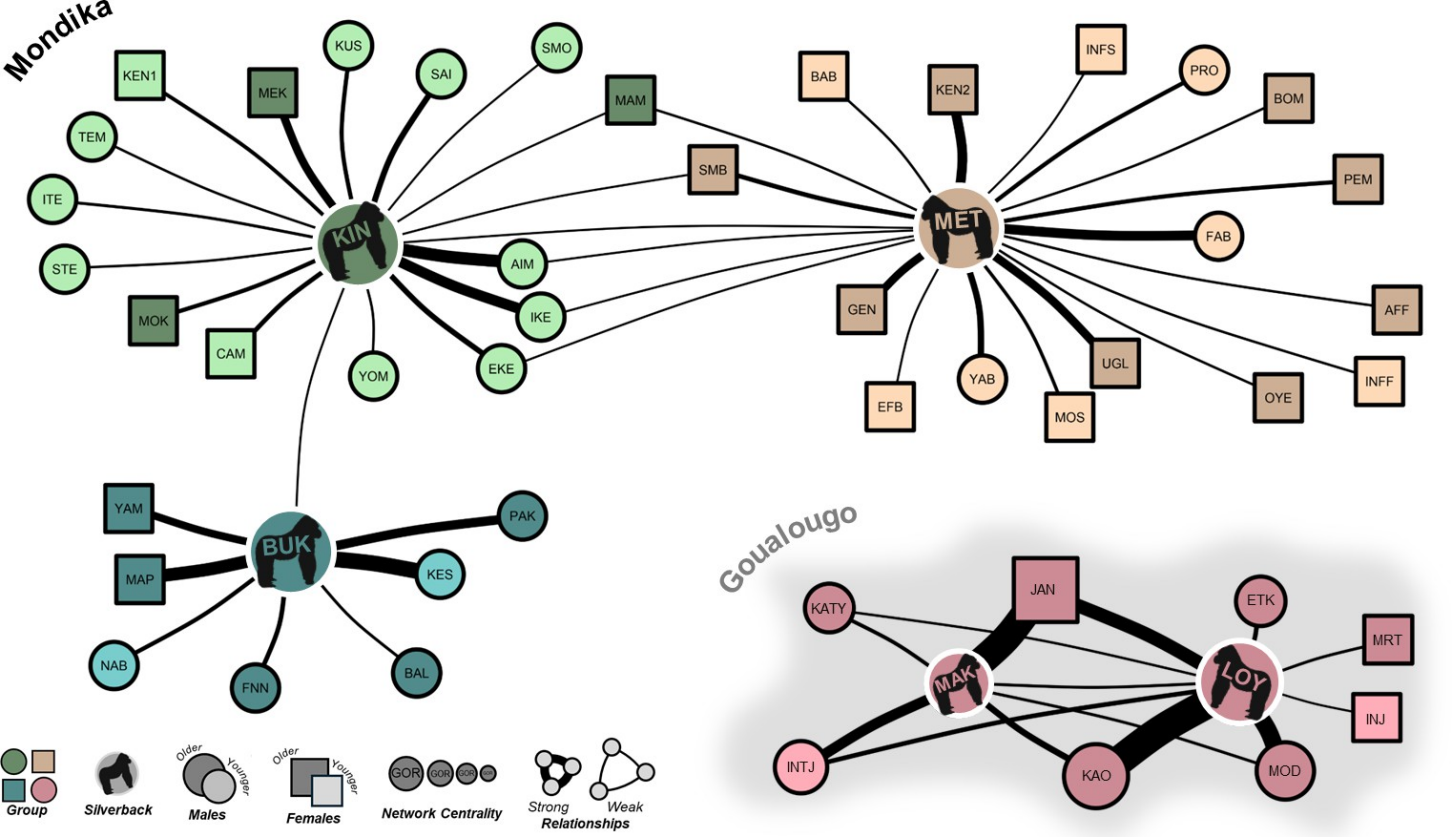
Silverback nearest neighbor networks for all study groups. Measured as simple ratio association indexes collected during group scans. All shapes (nodes) represent individual gorillas and the lines connecting them represent the relationships that they share. The width of the lines is scaled to relationship strength (simple ratio association index). The size of nodes and node labels (i.e., gorilla name) are scaled to within-group network centrality (an individual-level metric of network importance and influence: [60]), the color of the node indicates group membership. The shape of the node indicates sex, where females and immature individuals of unknown sex are squares and males are circles. Nodes are shaded to indicate average age during the study period, where the darkest are adults (males >11 years, females >10 years) and the lightest are immatures (i.e., infants and immatures). The silverback individuals are highlighted with gorilla silhouettes. The Loya-Makassa group resides in Goualougo (background shaded grey), whereas the neighboring Buka, Kingo, and Mététélé groups reside in Mondika. Silverback gorilla icons were created with BioRender.com and are reprinted under a CC BY license, with permission from BioRender, original copyright (2024), and used only for illustrative purposes.

The patterns of social interactions that we observed in this study indicate high social connectivity within and between groups. As described above, the overwhelming majority of the social interactions that composed this network (>99%) were affiliative in nature both within and between groups (also see *Table 2*). We did not observe any social relationships to be characterized entirely by aggression between any two individuals. The social interaction network is visualized in *Fig 3*. Additionally, the pattern of silverback nearest neighbor associations that we observed illustrates the frequency of occasions where silverbacks were the nearest neighbor of intergroup individuals (n = 105 observations), including silverbacks from other groups (n = 6 observations). The silverback nearest neighbor network is visualized in *Fig 4*. Further, results of bootstrapped paired-samples t-tests indicate that the observed social interaction network was composed of significantly higher connectivity (i.e., density including intergroup social relationships) than the constructed expected networks (observed density of present relationships [0.1243]–expected density [0.0671] = 0.0572; T = 3.07, 95% CI = 0.0207–0.0938, P = 0.0032). These results support the prediction that gorilla social relationships extend across neighboring groups, including between neighboring silverback males.

### Intragroup social relationships

Results of bootstrapped paired-samples t-tests of the observed social interaction network and the constructed simulated removal network for each age-sex class revealed that blackback and immature individuals significantly impact community connectivity (see *Table 5*). In all groups, the removal of all blackback individuals markedly decreased the number of intergroup connections (63% decrease) while the removal of immature individuals had the same effect on intra-group connections (66% decrease). Conversely, the removal of all silverback and adult female individuals did not significantly impact the network (22% decrease; 27% decrease respectively; see *Table 5*). These results indicate that intragroup and intergroup social relationships are predominantly influenced by non-adult individuals (i.e., immatures and blackback).

**Table 5:** Results of simulated network removals for all age and sex classes.

Network	Density	*t*-statistic [95% CI]	*p*-value
Observed all-occurrence social interaction network…			
~ Silverbacks simulated removal	3.0533, 2.3640	1.51 [-0.2028, 1.5812]	0.0822
~ Blackbacks simulated removal	3.0533, 1.0890	2.83 [0.6053, 3.3233]	0.0100
~ Adult females simulated removal	3.0533, 2.2099	1.85 [-0.0505, 1.7372]	0.0504
~ Immatures simulated removal	3.0533, 0.3653	3.34 [1.1080, 4.2680]	0.0020

Paired-sample, bootstrapped t-tests of the observed weighted social interaction network compared to networks constructed with simulated removals for each age-sex class. Tests were conducted with 5,000 iterations.

## Discussion

Our results provide novel insights into the extent of western lowland gorilla social relationships within and between neighboring groups. Affiliative interactions represent an overwhelming majority of western lowland gorilla social behavior, whereas we recorded aggression to be rare across all groups and individuals. Male gorillas were more social than females, younger individuals were more social than older, and slightly greater rates of social behaviors were observed during periods of higher fruit availability. The inter- and intragroup social relationships occurred at a higher density and connectivity than expected. Simulated removal of immatures and blackbacks from the social interaction network resulted in the greatest change in network density, a measure of overall connectedness. In previous investigations, neighboring groups of this species were occasionally tolerant of one another during intergroup encounters [18], but our longitudinal observations of the social behavior of these same groups are the first to be able to speak to the longstanding suggestion that males in neighboring groups are familiar with one another and likely share long-lasting social relationships. The existence and patterning of these intergroup social relationships could facilitate the emergence of communities of neighboring groups within a hierarchical social structure [19, 20]. These findings clarify aspects of gorilla sociality by highlighting variation in their social organization and contributing to conversations about the function of specific social behaviors and relationships. Importantly, such an enhanced understanding of gorilla society can provide insights for conservation strategies and the management of captive populations of these endangered apes.

Previously, it seemed that competition for ripe fruits might prohibit affinitive types of social relationships among western lowland gorillas [3–5]. However, we observed very little competitive or aggressive behavior overall and found that frequencies of social behavior (predominantly composed of play and other affiliative interactions) slightly increased with higher fruit availability. This suggests that a greater presence of fruit might foster opportunities for familiar individuals within and between groups to co-feed and engage in social interactions, as has been reported in other apes suggested to have multi-level societies (e.g., *Pan paniscus*: [68, 69]).

There has been a historical emphasis on the importance of silverbacks in intragroup and intergroup social dynamics. While competition for resources and mates are likely the primary motivations for silverback male behavior [70], we observed these males to exhibit a high degree of social tolerance both within and between groups. Silverbacks spent time in close proximity to all group members and engaged in play interactions with immature individuals in the group. Silverbacks from neighboring groups were also repeatedly observed to be tolerant nearest neighbors of one another. Rather than choosing to associate with any members of their own groups, Mététélé and Kingo, Kingo and Buka, and other individuals from different groups chose to be nearest to one another on occasion. Such an extended, repeated, and longitudinal expression of tolerance to other silverbacks likely facilitated the transition we observed during this study period, where Loya’s group transitioned from a one-male to a multi-male group. To our knowledge, this is the first confirmed report of a western lowland group that has remained consistently composed of more than one silverback for several years and that was not formed by the late dispersal of a mature, natal male.

While we had predicted that both intra- and intergroup social relationships would be centered around the silverback males, our combined approach of behavioral assessments with network-based approaches enabled us to examine the social contributions and connectedness of individuals across all age and sex classes. Our findings highlight the prominent role of immature individuals and blackback males within and across groups. Immature individuals demonstrated an important role in group connectedness, with social play comprising an overwhelming majority of this population’s social behavior. Social play has long been considered an important component of primate social behavior [71]. While the specific short- and long-term functions of play remain largely unknown for gorillas [72], social interactions and frequent proximity likely provide opportunities for the acquisition of cultural behaviors that are repeatedly transmitted via social learning, both within and across generations (for an overview of animal cultures, see [73]). While documenting specific social learning processes in the wild can be challenging, researchers have used population-level differences among sites with similar ecological opportunities to be indicative of putative cultural variants [74–76]. Western lowland gorillas live in heterogeneous environments that could favor social inheritance of particular foraging and/or social skills. Given the density of social relationships that we report here both within and between western gorilla social groups, and the potential opportunities for social learning that they represent, future work could assess whether behavioral variation correlates with patterns of social relationships within and between groups. Furthermore, we acknowledge that nuanced, longitudinal examinations of functional changes in social dynamics are difficult to model with static social network analyses. We look forward to future investigations that use dynamic, multidimensional, time series, and diffusion-based network analytics to account for changes in gorilla demographics and behavioral strategies that we were unable to examine in the current study. Such approaches offer a new understanding of gorilla societies.

Another implication of social associations and interactions is the risk of infectious disease transmission. It has been reported that the potential for within-group transmission of pathogens among mountain gorillas is relatively high [13] and could be even more elevated in densely connected networks of few individuals, as is typical of western lowland groups. In previous studies of the Nouabalé-Ndoki National Park gorilla groups, males displayed more respiratory symptoms than females, age was positively correlated to symptomatic observations, silverbacks showed a much higher prevalence of respiratory signs of illness than any other age-sex class, and there were increased clinical signs during periods of low fruit availability [14]. Previous investigations also highlighted the possible network effects of transmissibility, as the size of these groups was not a predictor of respiratory symptoms [14]. However, transmission events are difficult to detect, particularly if multiple neighboring social groups are not habituated (e.g., [77]). The Mondika research station offers a unique opportunity in central Africa to observe multiple habituated groups who navigate overlapping home ranges [22]. On one occasion at Mondika, the otherwise healthy members of Buka’s group were observed to exhibit clinical signs of respiratory illness days after interacting with members of Kingo’s group who were experiencing respiratory signs at the time [14]. Our findings of this investigation regarding the importance of immature and blackback gorillas in mediating densely connected networks of social relationships potentially add support to existing hypotheses that these age classes might disproportionately contribute to the transmission of information and pathogens within and between groups (e.g., [78]). Improving our understanding of the functions of social relationships and transmission pathways within and between groups is especially important to consider in managing both wild and captive gorilla populations.

Despite decades of research, it remains unclear if the social environment of captive western lowland gorillas adequately mirrors the dynamic social lives of their wild counterparts (e.g., [79]). Thousands of western lowland gorillas live in zoos and sanctuaries across the globe. The historic emphasis on a one-male social structure of this species has in part led to a surplus of captive males who are usually housed in all-male bachelor groups for the beginning or all of their adult lives [79–82]. Our observations of a naturally occurring group with more than one silverback for several years and tolerant intergroup interactions might foster conversations about the future of bachelor and multimale-multifemale western lowland gorilla groups in captive environments. Since this study period, we observed Kingo and Buka groups evolve into all-male bachelor groups as the age of each silverback became more advanced. Our observations of these gradual changes to the wild groups’ compositions might prompt consideration of forming bachelor groups for males of advanced age in managed settings. Information about affiliative inter- and intragroup social behaviors, particularly among silverbacks, can also be used to inform captive gorilla management and wellbeing. In addition, our findings about the highly social nature of blackbacks and maturing young silverbacks support the growing occurrence of captive gorilla group introductions featuring unrelated individual males. More specifically, there may be an early developmental stage as well as a later-in-life window of opportunity for integrating unrelated males into established groups. We hope that our investigation can provide a useful reference point to compare rates of social interactions across individuals and age classes within and between captive groups as a measure of social wellbeing.

The critically endangered status of western gorilla populations makes their study and conservation an urgent matter. In the face of habitat degradation, anthropogenic disturbance, climate change, and infectious diseases [83, 84], it is necessary to develop proactive strategies to address the risks they face. Gorilla-focused tourism is one conservation strategy that can provide alternative streams of revenue for local communities that also has the potential to aid in countering illegal hunting activities and removal of forest resources from remaining ape habitats. As with any type of anthropogenic pressure, it will be important to ensure that the impact of tourism on the gorillas and their habitats is minimized and does not cause adverse effects. In light of the current efforts to facilitate the growth of tourism across the region, the results described here will serve as a baseline to monitor within-group changes in relation to visitor presence and proximity which are factors shown to impact gorilla sociality [78, 85]. There are also risk factors associated with increasing human activity in gorilla-populated landscapes [10, 86, 87]. In response to concerns about disease transmission, standardized protocols have been developed to reduce the risk of disease transmission from humans and also to conduct observational health assessments of wild apes. Another conservation initiative involves coordinating such monitoring across sites so as to gain landscape-scale perspectives on health, ecology, and behavior. Towards achieving this goal, standardized protocols and methods for collecting observational health assessments, behavioral, and ecological data on habituated gorillas has been implemented across World Heritage sites including the Nouabalé-Ndoki National Park (Goualougo, Mondika stations) and the Dzanga-Sangha Protected Area (Mongambe and Bai Houkou stations) of the Sangha Trinational Region and the Odzala-Kokoua National Park (Imbalanga station), and part of the Trinational Dja-Odzala-Minkébé Area (TRIDOM) Landscape. All of these coordinated efforts will increase the effectiveness of our ability to detect and mitigate the threats that these wild apes share, while also resulting in an expanded rubric of conservation indicators that could include networks of social relationships and cultural repertoires that deserve conserving on their own merit [76, 88]. In conclusion, we hope that our findings inspire broader consideration of the extent of social connections within and between groups on gorilla survival and wellbeing in both wild and captive environments.

## Supporting information

S1 Checklist Inclusivity in global research. (DOCX)
